# Selecting Effective Herbal Medicines for Attention-Deficit/Hyperactivity Disorder via Text Mining of Donguibogam

**DOI:** 10.1155/2019/1798364

**Published:** 2019-06-04

**Authors:** Hyo Won Bae, Se Yeon Lee, Sung Ji Kim, Hwa Kyoung Shin, Byung Tae Choi, Jin Ung Baek

**Affiliations:** ^1^Division of Humanities and Social Medicine, School of Korean Medicine, Pusan National University, Yangsan 626-870, Republic of Korea; ^2^Division of Meridian and Structural Medicine, School of Korean Medicine, Pusan National University, Yangsan 626-870, Republic of Korea

## Abstract

**Objective:**

Several attempts have been made to reduce the harmful side effects and increase the efficacy of current drugs used to treat attention-deficit/hyperactivity disorder (ADHD). Many articles have studied medicinal herbs as an effective supplement in treating ADHD. In a similar manner, this study provides foundational data to identify herbs that are potentially effective in treating ADHD by text mining of Donguibogam, which is a comprehensive summation of the important traditional principles and practices of Korean medicine.

**Methods:**

Text mining was performed for 3833 herbal prescriptions and 1108 medicinal herbs comprising prescriptions listed in Donguibogam. The first step was frequency analysis followed by chi-square test, which is a statistical hypothesis test.

**Results and Conclusions:**

Twelve medicinal herbs were selected for each ADHD subtype: hyperactivity ADHD type (ADHD-PHI) and attention-deficit ADHD type (ADHD-PI). Compared to previous research on traditional literature, a newer and more efficient methodology of selecting herbal medicines was developed in this process.

## 1. Introduction

Patients with attention-deficit/hyperactivity disorder (ADHD) present with symptoms such as lack of concentration, impulsiveness, and excessive activity, and ADHD is one of the neurobehavioral disorders commonly occurring in childhood. [1].

Currently, approximately 5-7% of school-aged children and 2-5% of adults are believed to have ADHD worldwide [2, 3]. In the United States, the percentage of ADHD patients among children and adolescents was 6.1% (Diagnostic and Statistical Manual of Mental Disorders [DSM]-IV) in 1998 and 10.2% (DSM-V) in 2016 [4]. Some argue that with the development of diagnostic methods, the number of ADHD patients in the United States increased over the years, and a similar increase occurred in many European countries [5–7]. In Korea, a less than 1% diagnostic rate was reported in a study using actual hospital treatment records, but 5.9% of children with ADHD were reported in another study using the random sampling method in elementary school students [8, 9]. This indicated that although a sizable portion of the population experienced ADHD symptoms, they have not visited the hospital for diagnosis.

Both genetic and environmental factors affect the occurrence of ADHD, but the exact cause has not yet been elucidated. However, some researchers have found that symptoms of impulsiveness and attention-deficiency tend to be present when the function of the frontal lobe of controlling thought and concentration decreases. Another possible reason could be a problem in metabolizing neurotransmitters such as catecholamine, norepinephrine, and dopamine [10]. Therefore, stimulants of the central nervous system (methylphenidate and pemoline), norepinephrine reuptake inhibitors (atomoxetine), or antidepressants are mainly used as treatment drugs [11]. Approximately 90% of Korean ADHD patients were prescribed methylphenidate, because drugs approved for use in Korea are limited and pemoline has been linked to hepatotoxicity [12]. However, side effects of methylphenidate include decreased appetite, insomnia, growth delay, tic disorder, nausea, stomachache, headache, and fatigue [13, 14]. In preschool children below 7 years of age in particular, 30% of them were more likely to suffer growth delay compared to school-age children, which meant that they experienced more severe side effects [15]. To avoid such harmful side effects, natural and holistic treatments using Korean medicine need to be considered as an alternative.

Thus far, traditional Korean medicine or traditional Chinese medicine research related to ADHD has been mainly composed of research based on original literature, systematic review based on search treatises, and experimental research testing the treatment effect of herbs on ADHD [16–21]. However, research based on original literature was limited to only listing herbal medicines that frequently appeared in text. On the other hand, experimental research had difficulty in selecting the right herbal medicines to test. To overcome these limitations, this study preselected a list of herbal medicines that statistically significantly deemed potentially effective for ADHD by screening Donguibogam, which is a comprehensive compilation of important traditional Eastern medicines. This study played the role of a preliminary experiment to not only save time and money, but also increase the success rate of future experimental research.

Furthermore, this study considered hyperactivity ADHD type (ADHD-PHI) and attention-deficit ADHD type (ADHD-PI) as two separate disease symptoms. DSM is the most typical diagnostic method of ADHD, which classifies ADHD into three subtypes: predominantly inattentive (ADHD-PI), predominantly hyperactive-impulsive (ADHD-PH), and combined type (ADHD-C) [22]. In Western medicine, symptoms of ADHD are heavily linked to the frontal lobe of the brain or neurotransmitters, and the same drugs (such as central nervous system stimulants, norepinephrine reuptake inhibitors, or antidepressants) are used to treat both ADHD-PI and ADHD-PHI. However, traditional Korean medicine uses symptomatic treatment that pays close attention to each symptom. Therefore, this study found medicinal herbs specific for each subtype (ADHD-PHI and ADHD-PI) and also listed herbs that could be applied to both.

## 2. Materials and Methods

The herbal prescriptions deemed potentially effective for ADHD in Donguibogam (ADHD PEHP) were compared to the remaining herbal prescriptions in the book using the chi-square test, a statistical method used to determine homogeneity between two groups for a specific variable. Thus, the authors were able to select only those herbs showing statistically significant efficacy.

### 2.1. Generating Baseline Data for Analysis

First, all prescriptions in Donguibogam were extracted, and the herbs comprising each prescription were listed. In this process, medicinal herbs with synonymous names were standardized under one name.

**Figure 1:**
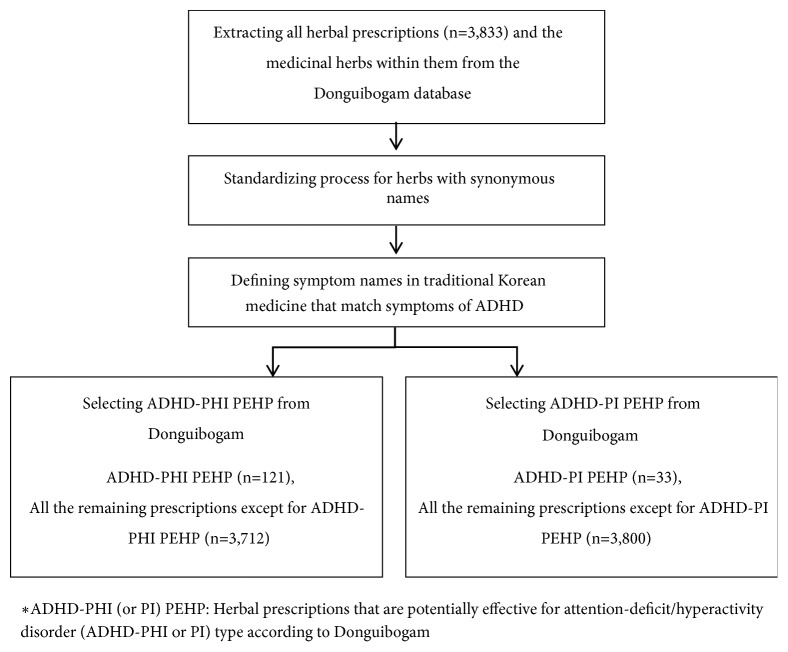
Selecting herbal prescriptions deemed potentially effective for attention-deficit/hyperactivity disorder in Donguibogam (ADHD PEHP).

Second, Korean medical symptom names corresponding to ADHD-PHI and ADHD-PI were defined using existing research, and prescriptions and herbs containing at least one of these symptom names were selected (Figure 1) [23]. Information regarding prescriptions and herbs in Donguibogam was extracted from a state-run website called the Korean traditional knowledge portal (http://www.koreantk.com/ktkp2014).

*Symptom Names in Traditional Korean Medicine That Matched ADHD*

- ADHD-PHI: Jingji, zhengchong, zhanyu, xu fan, fan zao, yin xuhuo dong, and shanghan xu xuezheng (searched by Xu Xue)
- ADHD-PI: Jianwang, laojuan shang, qi fa, and yin xu neire

### 2.2. Statistical Analysis

The frequency of occurrence was calculated for each medicinal herb in ADHD-PHI PEHP. Only those herbs that made up more than 2% of the total were selected as the experimental group (Table 1).

**Figure 2:**
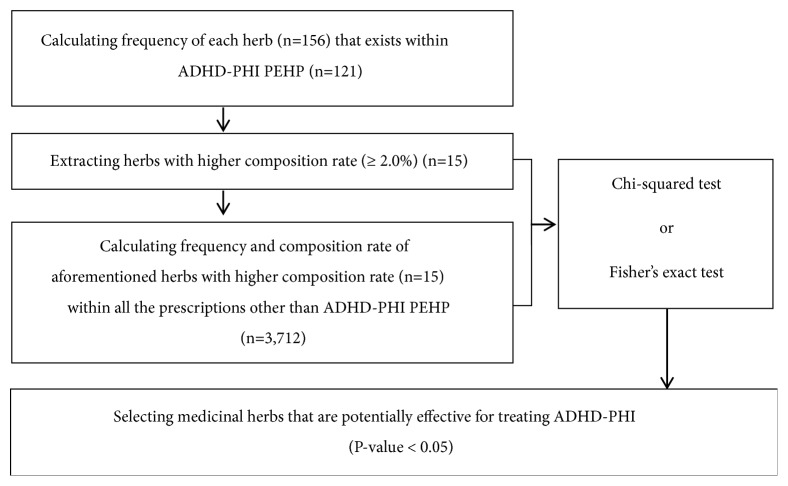
Statistical stage of selecting herbs deemed potentially effective for hyperactivity attention-deficit/hyperactivity disorder type.

**Figure 3:**
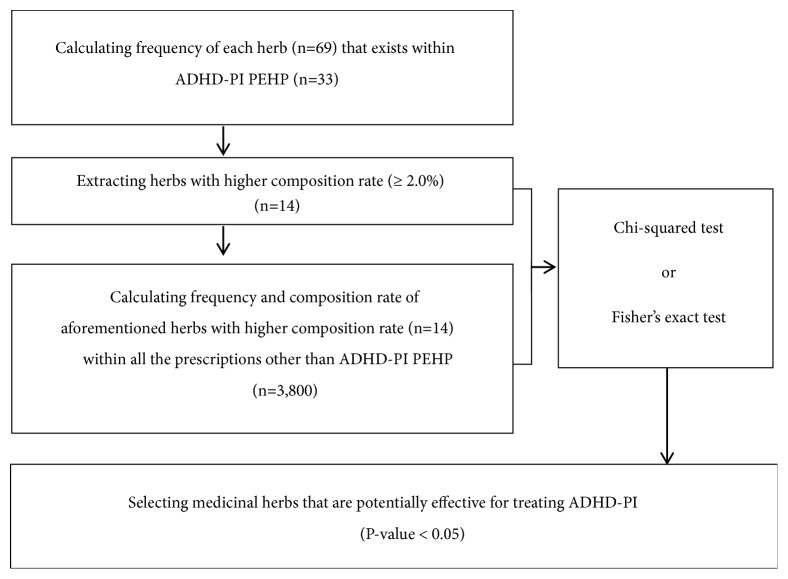
Statistical stage of selecting herbs deemed potentially effective for attention-deficit attention-deficit/hyperactivity disorder type.

Among these, “Cinnabaris” was removed because it is a medicinal herb derived from minerals and its use is restricted. Additionally, the control group was selected by counting the number of times the herbs that were selected as the experimental group appeared in the rest of the Donguibogam prescriptions, except for ADHD-PHI PEHP. The same steps were repeated for the prescriptions potentially effective for ADHD-PI. Following this, a chi-squared test was performed to evaluate whether experimental group herbs were more effective in the treatment of ADHD-PHI or ADHD-PI in a statistically significant manner than control group herbs (Figures 2 and 3) (P < 0.05). However, in the chi-squared test, Yate's continuity correction was used when the expected frequency was 5-9, and Fisher's exact test was used when it was under 5 for more than 20% of the cells [24]. For data analysis, Microsoft Excel 2016 was used to generate baseline data, and SAS version 9.4 was used for statistical analysis.

## 3. Results

### 3.1. ADHD-PHI

**Figure 4:**
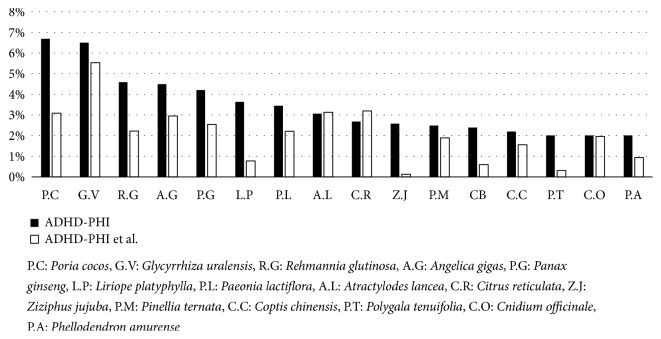
Comparison of composition rate for herbs deemed potentially effective for hyperactivity attention-deficit/hyperactivity disorder type (ADHD-PHI).

For symptoms of ADHD-PHI, the top 5 herbs with the highest composition rate were* Poria cocos* (P.C, 6.69%),* Glycyrrhiza uralensis* (G.V, 6.50%),* Rehmannia glutinosa* (R.G, 4.59%),* Angelica gigas* (A.G, 4.49%), and* Panax ginseng* (P.G, 4.21%) (Figure 4).

Compared to the rest of the prescriptions, other than those of ADHD-PHI PEHP, the usage of P.C, G.V, R.G, A.G, P.G,* Liriope platyphylla* (L.P),* Paeonia lactiflora* (P.L),* Ziziphus jujuba* (Z.J),* Coptis chinensis* (C.C),* Polygala tenuifolia* (P.T), and* Phellodendron amurense *(P.A) for ADHD-PHI was statistically significant at 0.01 and that of* Pinellia ternata* (P.M) was significant at 0.05. The usage of* Atractylodes lancea* (A.L),* Citrus reticulata* (C.R), and* Cnidium officinale* (C.O) was not statistically significant for ADHD-PHI. In cases of L.P and C.O, Yate's continuity correction chi-squared value was used, while the Fisher's exact test was used for Z.J and P. T. Based on their chi-squared values, L.P (127.12), P.C (83.29), and R.G (47.41) were found to have been used specifically for ADHD-PHI (in that order of specificity). The chi-squared values of Z.J (349.09) and P.T (102.80) were very high, but the authors decided to exclude them from the ranking order of significance because 25% of the cells had an expected frequency lower than 5 (Table 2).

### 3.2. ADHD-PI

**Figure 5:**
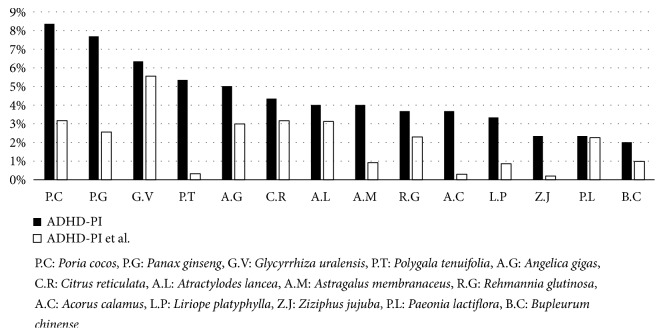
Comparison of composition rate for herbs deemed potentially effective for attention-deficit attention-deficit/hyperactivity disorder type (ADHD-PI).

For symptoms of ADHD-PI, the top 5 herbs with the highest composition rate were P.C (8.36%), P.G (7.69%), G.V (6.35%), P.T (5.35%), and A.G (5.02%) (Figure 5).

Compared to the rest of the prescriptions, other than those of ADHD-PI PEHP, the usage of P.C, P.G, P.T, A.G, A.M, R.G, A.C, L.P, and Z.J for ADHD-PI was statistically significant at 0.01 and that of G.V, C.R, and B.C was significant at 0.05. The usage of A.L and P.L was not statistically significant for ADHD-PI. For P.C, P.G, A.G, C.R, A.L, R.G, and P.L, Yate's continuity correction chi-squared value was used, while the Fisher's exact test was used for P.T, A.M, A.C, L.P, Z.J, and B.C. Based on their chi-squared values, P.G (52.90) and P.C (48.26) were found to have been used specifically for ADHD-PI. The chi-squared values of P.T (266.99), A.C (137.65), and Z.J (83.39) were high, but the authors decided to exclude them from the ranking order of significance because Fisher's exact test was used (Table 3).

## 4. Discussion

By reviewing the results of the analysis, 12 out of the 156 herbs comprising the ADHD-PHI PEHP and 12 out of the 69 herbs comprising the ADHD-PI PEHP were determined to be significant for further experiments. Figures 4 and 5 showed that the statistical significance tended to present more clearly when considering the same medicinal herb, and the difference in composition rates was large between ADHD PEHP and the remaining prescriptions included in the 3833 prescriptions in Donguibogam. Moreover, when the difference in composition rates was too close to be determined, statistical tests became a useful standard of judgement. For example, G.V and P.M of ADHD-PHI and G.V and C.R of ADHD-PI were included in the list of potential herbs for ADHD treatment. On the other hand, A.L, C.R, and C.O of ADHD-PHI and A.L and P.L of ADHD-PI, which would have been selected in previous studies using simple frequency counting, were excluded.

The authors presented the order of importance of selected herbal medicines using statistics (chi-squared values). The aforementioned points indicated that the methodology of this study was more systematic and efficient in selection.

To reflect the characteristic of symptomatic treatment in Korean medicine, the study was conducted separately for ADHD-PHI and ADHD-PI. The results show that 8 herbs (P.C, G.V, P.G, P.T, A.G, R.G, L.P, and Z.J) were selected with statistical significance for both subtypes of ADHD. However, 4 herbs (P.L, P.M, C.C, and P.A) were significant only for ADHD-PHI, while the other 4 (C.R, A.M, A.C, and B.C) were significant only for ADHD-PI. For each subtype of ADHD, there were 4 herbs that did not apply to the other subtype, which validated the approach in Korean medicine to consider different symptoms as separate diseases.

Since this study was an analysis of original literature, its limitation was that it needed experimental verification. However, this study remains insightful by presenting a more efficient text mining method using statistical analysis. This may be the stepping stone in the future to utilize more diverse statistical tools of analysis of original research in Korean medicine.

## 5. Conclusions

P.C, G.V, R.G, A.G, P.G, L.P, P.L, Z.J, P.M, C.C, P.T, and C.O were selected as herbs showing a high probability of effectively treating ADHD-PHI. Simultaneously, P.C, P.G, G.V, P.T, A.G, C.R, A.M, R.G, A.C, L.P, Z.J, and B.C were selected as herbs with a high probability of effectively treating ADHD-PI.

Furthermore, this study presented the chi-squared test as a new method for obtaining medicinal herbs that are useful for treating ADHD.

## Figures and Tables

**Table 1:** Medicinal herbs selected as the experimental group (n=18).

Name	Author	Part of the plant	Abbreviation
*Acorus calamus*	L.	rhizoma	A.C

*Angelica gigas*	Nakai	root	A.G

*Atractylodes lancea*	(Thunb.) DC.	rhizoma	A.L

*Astragalus membranaceus*	(Fisch.) Bunge	root	A.M

*Bupleurum chinense*	DC.	root	B.C

*Coptis chinensis*	Franch.	rhizoma	C.C

*Cnidium officinale*	Makino	rhizoma	C.O

*Citrus reticulata*	Blanco	peel	C.R

*Glycyrrhiza uralensis*	Fisch.	root	G.V

*Liriope platyphylla*	F.T.Wang et Tang	tuber	L.P

*Phellodendron amurense*	Rupr.	cortex	P.A

*Poria cocos*	Wolf		P.C

*Panax ginseng*	C.A.Mey.	root	P.G

*Paeonia lactiflora*	Pall.	root	P.L

*Pinellia ternata*	(Thunb.) Makino	tuber	P.M

*Polygala tenuifolia*	Willd.	root	P.T

*Rehmannia glutinosa*	(Gaertn.) DC.	root	R.G

*Ziziphus jujuba var. spinosa*	(Bunge) Hu ex H.F.Chow	seed	Z.J

**Table 2:** The results of chi-squared test for herbs deemed potentially effective for hyperactivity attention-deficit/hyperactivity disorder type (ADHD-PHI).

Herb name	P.C^1^	G.V^2^	R.G^3^	A.G^4^	P.G^5^	L.P^6^	P.L^7^	A.L^8^	C.R^9^	Z.J^10^	P.M^11^	C.C^12^	P.T^13^	C.O^14^	P.A^15^
*χ* ^2^	83.29	13.16	47.41	21.42	25.01	127.12	16.47	1.03	0.00	349.09	6.15	7.03	102.8	1.06	18.53

P-value	**<.0001**	**0.0003**	**<.0001**	**<.0001**	**<.0001**	**<.0001**	**<.0001**	0.3101	0.9614	<.0001	**0.0131**	**0.008**	<.0001	0.3024	**<.0001**

Fisher										**<.0001**			**<.0001**		

^1^ *Poria cocos.*

^2^ *Glycyrrhiza uralensis.*

^3^ *Rehmannia glutinosa.*

^4^ *Angelica gigas.*

^5^ *Panax ginseng.*

^6^ *Liriope platyphylla.*

^7^ *Paeonia lactiflora.*

^8^ *Atractylodes lancea.*

^9^ *Citrus reticulata.*

^10^ *Ziziphus jujuba.*

^11^ *Pinellia ternata.*

^12^ *Coptis chinensis.*

^13^ *Polygala tenuifolia.*

^14^ *Cnidium officinale.*

^15^ *Phellodendron amurense*.

**Table 3:** The results of chi-squared test for herbs deemed potentially effective for attention-deficit attention-deficit/hyperactivity disorder type (ADHD-PI).

Herb name	P.C^16^	P.G^17^	G.V^18^	P.T^19^	A.G^20^	C.R^21^	A.L^22^	A.M^23^	R.G^24^	A.C^25^	L.P^26^	Z.J^27^	P.L^28^	B.C^29^
*χ*2	48.26	52.9	4.14	266.99	9.62	4.19	2.83	45.33	5.47	137.65	32	83.39	0.28	6.11

P-value	**<.0001**	**<.0001**	**0.0419**	<.0001	**0.0019**	**0.0407**	0.0925	<.0001	**0.0193**	<.0001	<.0001	<.0001	0.5994	0.0135

Fisher				**<.0001**	** **	** **	** **	**<.0001**	** **	**<.0001**	**<.0001**	**<.0001**	** **	**0.0272**

^**16**^ *Poria cocos.*

^**17**^ *Panax ginseng.*

^**18**^ *Glycyrrhiza uralensis.*

^**19**^ *Polygala tenuifolia.*

^**20**^ *Angelica gigas.*

^**21**^ *Citrus reticulata.*

^**22**^ *Atractylodes lancea.*

^**23**^ *Astragalus membranaceus.*

^**24**^ *Rehmannia glutinosa.*

^**25**^ *Acorus calamus.*

^**26**^ *Liriope platyphylla.*

^**27**^ *Ziziphus jujuba.*

^**28**^ *Paeonia lactiflora.*

^**29**^ *Bupleurum chinense*.

## Data Availability

The data used to support the findings of this study are included within the article.
